# Psychosocial factors at work and their impact on health: contributions to current debates

**DOI:** 10.47626/1679-4435-2025-231

**Published:** 2025-08-25

**Authors:** Frida Marina Fischer, Maria Carmen Martinez

**Affiliations:** Full Professor, Department of Environmental Health, University of São Paulo, School of Public Health; Epidemiologist and Independent Consultant, WAF Informatics & Health

The changes in Norma Regulamentadora 1 (NR-01), “General Provisions and Occupational Risk Management” (effective May 25, 2025), provide for the inclusion of psychosocial factors in occupational risk management.^1^ This can be largely attributed to the increased incidence of stress-related and mental health problems resulting from exposure to negative factors in the work environment and their consequent impact on workers, institutions and health, social security, and pension systems.

Exposure to psychosocial factors at work has been increasing over the last few decades as a result of unfavorable changes in work conditions and organization.

Interest and studies on psychosocial factors at work grew throughout the 20th century, including authors such as Elton Mayo (1920s - School of Human Relations), Frederick Herzberg (1950s - Theory of Hygiene and Motivational Factors influencing satisfaction), Cooper & Marshall (1970s - Occupational Stress), Karasek & Theorell (1970s - Demand-Control Model, and Johannes Siegrist (1990s - Effort-Reward Model).^2^,^3^ Swedish author Lennart Levi was also relevant, founding the Stress Research Laboratory in 1959 and the National Institute for Psychosocial Factors and Health in 1980.^4^ Among his many works, he authored *The Psychosocial Environment and Psychosomatic Diseases*, part of the Society, Stress, and Disease series,^5^ as well as *Guidance on work-related stress: Spice of life or kiss of death?*^6^

This special issue of Revista Brasileira de Medicina do Trabalho considers psychosocial factors at work through different methodological approaches. Below we will briefly comment on some of the most relevant aspects of the articles.

In an opinion article, Lucca, Silva-Junior, and Bandini describe historical aspects related to World Health Organization and the International Labour Organization guidelines and recommendations from 1984 to the most recent changes in Brazilian labor legislation. The authors draw attention to the asymmetrical relationships in capital-labor relations and the need to observe collective dimensions when evaluating psychosocial factors at work. They highlight the need for a systemic approach to work environments and the psychosocial factors they entail, as well as appropriate use of health-focused management models.

Rocha et al. present a brief review of the main recognized phases of work organization from the time of Frederick Taylor until the current “performance society” and its forms of production management. They discuss the negative repercussions associated with the psychological demands of precarious and often embarrassing work. They also describe the concepts of performativity and burnout syndrome and their relationships with psychosocial factors at work.

Four of the nine articles in this special issue are about health care workers. This can be explained by the way work is organized in this sector, with numerous differences and nuances between labor categories, but always in conditions that can harm the health of those who care for the health of others.

Oliveira et al. present an assessment of psychosocial factors at work and their contribution to illness among ICU nurses. An integrative literature review found that ICU nurses have increased risks due to the sector’s dynamics, complexity, and specialty, with workloads that produce stress, suffering, illness and compromise professional performance. They point out that the intensification of psychosocial factors at work has led to negative outcomes in recent decades, as well as the lack of studies with more robust designs, such as longitudinal investigations that show causal relationships and use validated assessment instruments. They also point out the gap between knowledge production and its use in preventive interventions.

In a descriptive study, Duarte et al. evaluated psychosocial factors at work among registered and practical nurses in the ICU of a general hospital in midwestern Brazil. The workers were exposed to medium-level risk for factors inherent to the division of tasks, a lack of recognition, and psychological, social, and physical harm. High risks inherent to the social division of labor and to mental exhaustion were observed. Interventions were suggested to improve communication and relationships between teams, to prevent prejudice, discrimination, and violence in the work context, to improve work organization and conditions, and to improve free time/leisure, sleep, and diet.

In a general hospital, Vale et al. investigated psychosocial factors at work and the prevalence of psychological distress among workers and health professionals. Standardized instruments and interviews with workers were used to collect data. A high prevalence of common mental disorders was identified, in addition to an association between stressors and psychological distress. One possible cause for this was an increase in the number of hospital visits after regionalization, which resulted in increasing physical and mental demands that were probably associated with insufficient staff and equipment. Several dimensions helped regulate the mental health of these workers. While the increased demands and hostile communication between managers and subordinates were negative factors, identifying with the work and the meaning attributed to care were positive factors, producing symbolic and identity-based rewards.

In a cross-sectional study, Ortiz-Chamorro sought to identify psychosocial factors that were both internal and external to work among recently graduated Colombian doctors who worked in mandatory social services during the COVID-19 pandemic. There was a relevant frequency of very high-intensity internal and external psychosocial risks, which aligned with the results of other Colombian studies. These risks could be associated with intense stress responses related to emotions and feelings. Intervention strategies are called for in an epidemiological surveillance system.

Identifying psychosocial factors at work is not the task of a specific professional, but requires the participation of actors from different professional categories, both management and employees, through different diagnostic and intervention strategies. Pereira’s opinion article reflects on how the Historical-Cultural Clinical Theory can interface with worker health to address psychosocial risks at work. To this end, the author presents an action-oriented method based on a questionnaire to qualitatively assess psychosocial risks at work and their relationship with worker health. The method seeks to identify needed changes, intervene in the social structure, and produce regulations to promote decent working conditions.

In a cross-sectional study, Rodrigues investigated the relationship between psychosocial factors at work and musculoskeletal symptoms among university professors. The new adverse scenarios these professionals face are discussed. An assessment of musculoskeletal symptoms, their functional repercussions, and their associations with psychosocial stressors in the work context is presented. Modeling based on structural equations is used to analyze factors associated with the observed effects. The author emphasizes the importance of risk factor management to prevent and mitigate damage to the health of professors.

Using qualitative approaches, Lopes & Lucca investigated five industrial companies in São Paulo state. They observed that work processes were mainly Taylorist, with rigid hierarchical management and poorly trained leaders. Even in companies that use more complex technology, assembly lines with strict time control predominated. Negative psychosocial factors at work were observed in all of the companies, such as low autonomy and little control over work, limited social support, limited or no career plans or recognition, psychological violence, and harassment.

Although the studies in this special issue have consistently identified high frequencies and intensities of exposure to psychosocial factors at work, as well as their harmful effects, especially on mental health, intervention studies that support good prevention practices, institutional actions, and public policies are still lacking.

The current context of the world of work is concerning, requiring urgent, continuous, systematic strategies based on best practices to control and prevent psychosocial factors at work. Despite its limitations, the new NR-01 provides an opportunity to reflect and act toward better work conditions and organization that promote worker health, well-being, and employability.
